# Polygenic Risk Score and Risk Factors for Gestational Diabetes

**DOI:** 10.3390/jpm12091381

**Published:** 2022-08-26

**Authors:** Marija Majda Perišić, Klemo Vladimir, Sarah Karpov, Mario Štorga, Ali Mostashari, Raya Khanin

**Affiliations:** 1 Faculty of Mechanical Engineering and Naval Architecture, University of Zagreb, 10000 Zagreb, Croatia; 2 LifeNome Inc., New York, NY 10018, USA; 3 Faculty of Electrical Engineering and Computing, University of Zagreb, 10000 Zagreb, Croatia; 4 Bioinformatics Core, Memorial Sloan-Kettering Cancer Center, New York, NY 10065, USA

**Keywords:** gestational diabetes, pregnancy, polygenic risk score, gwas, machine learning

## Abstract

Gestational diabetes mellitus (GDM) is a common complication of pregnancy that adversely affects maternal and offspring health. A variety of risk factors, such as BMI and age, have been associated with increased risks of gestational diabetes. However, in many cases, gestational diabetes occurs in healthy nulliparous women with no obvious risk factors. Emerging data suggest that the tendency to develop gestational diabetes has genetic and environmental components. Here we develop a polygenic risk score for GDM and investigate relationships between its genetic architecture and genetically constructed risk factors and biomarkers. Our results demonstrate that the polygenic risk score can be used as an early screening tool that identifies women at higher risk of GDM before its onset allowing comprehensive monitoring and preventative programs to mitigate the risks.

## 1. Introduction

Gestational diabetes mellitus (GDM) is a common complication of pregnancy that adversely affects maternal and offspring health. It is characterized by the onset of abnormal blood sugar (hyperglycemia) during pregnancy, typically in the second trimester, and is the most prevalent metabolic complication in pregnancy globally [1].

Diagnostic criteria for GDM differ by region and are largely influenced by conventional care and the preferences of the clinicians. The lack of uniformity in diagnosing GDM makes it difficult to accurately estimate its global prevalence. However, recent reviews concluded that GDM is most prevalent in the Middle East and North Africa (15.2%, 8.8–20.0% [median, interquartile range]) and South-East Asia (15.0%, 9.6–18.3%). The prevalence is lowest in North America and the Caribbean (7.0%, 6.5–11.9%) and Europe (6.1%, 1.8–31.0%), though the rates among European countries vary widely [2]. According to other sources, GDM is affecting up to 12–18% of all pregnancies [3].

GDM is usually discovered late in the second or early in the third trimester and refers to high blood sugar (glucose) during pregnancy. Women with a history of GDM have a 7-fold higher risk of developing type 2 diabetes (T2D) during midlife and an elevated risk of developing hypertension and cardiovascular disease [4]. It is therefore important to develop an early screening tool for identifying at-risk women to offer them comprehensive monitoring and preventative programs to mitigate the risks.

While high pre-pregnancy body mass index (BMI) accounts for about 41% of GDM cases for all ethnic groups, the remaining fraction of cases occur in healthy nulliparous women with no obvious risk factors. The reduced level of physical activity during pregnancy is partly responsible for the pregnancy-associated decline in metabolic health [5,6]. Hence, GDM is believed to be a result of interactions between genetic, epigenetic factors, advancing maternal age, and modifiable lifestyle factors [3,7] such as pre-pregnancy BMI, as well as physical activity and dietary intakes before and after conception [8,9,10].

Previous large-scale genome-wide association studies (GWAS) of GDM conducted across diverse populations have demonstrated association of genetic susceptibility to GDM with type 2 diabetes, insulin secretion and insulin resistance [11,12] suggesting a partial similarity of the genetic architecture behind the two forms of diabetes. Other GWAS, focusing on maternal metabolism during pregnancy, have demonstrated an overlap in the genes associated with metabolic traits in gravid and non-gravid populations, as well as in genes apparently unique to pregnancy [3,13].

Several genetics-based risk scores (polygenic risk scores, PGS) for GDM have already been published. The procedure to building these PGS generally starts with a preselected list of SNPs that have been found to be associated with either GDM, T2D, elevated fasting glucose and insulin, or reduced insulin secretion and sensitivity [14]. These SNPs are combined in a linear risk score model that generally shows significant associations with incidences of GDM but have limited predictive power for identifying GDM cases without clinical parameters. For example, PGS constructed from risk variants across 34 loci associated with T2D and fasting glucose was significantly associated with GDM in a study of Caucasian women that included 458 cases of GDM and 1538 pregnant controls with normal glucose tolerance. This PGS showed limited utility in the identification of GDM cases, only slightly improving predictive power over a model that includes only clinical variables [15].

Another case-control study that included 2636 women with GDM and 6086 controls, pre-selected a total of 112 SNPs related to T2D susceptibility, further identified 11 SNPs significantly associated with GDM, and used them to build a PGS which was significantly associated with a higher risk of GDM. Specifically, compared with participants in the lowest quartile of the PGS, the odds ratio for GDM in the highest quartile was 1.53 (95% CI = [1.34, 1.74]) [16]. A recent small study of Chinese women (475 cases and 487 controls) [17] built a PGS using 4 loci significantly correlated with the incidence of GDM. Authors report that genetic risk score was independently associated with GDM and was the most effective predictor with the exception of family history of diabetes. Combined with 6 clinical characteristics (maternal age, gravidity, parity, BMI, family history of diabetes and assisted reproduction) the new risk score has a good predictive power with the AUC of the prediction model was 0.727.

To untangle the genetic basis of GDM, we turned to the UK Biobank (UKBB), with the goal to develop a PGS for GDM using machine learning. We further set out to systematically investigate relationships between genetically constructed risk factors and GDM using Mendelian Randomization (MR).

## 2. Materials and Methods

### 2.1. Participants

This study utilizes the data of UKBB www.ukbiobank.ac.uk (accessed on 5 March 2022) which is a prospective cohort of 502,637 people aged between 37 and 73 and recruited from 2006 to 2010 from across the UK. The participants’ medical, socio-demographic, lifestyle, environmental, and genetic information was collected via detailed questionnaires and clinical assessment and linked with hospital admission and mortality data. The analysis reported in this paper included 273,309 UKBB participants self-identifying as females, for which no mismatch between self-reported and genetic gender was detected.

All procedures and data collection in UKBB were approved by the UKBB Research Ethics Committee (reference number 11/NW/0274), with participants providing full written informed consent for participation in UKBB and subsequent use of their data for approved applications.

To identify gestational diabetes cases, we retrieved information from touchscreen questionnaire “Did you only have diabetes during pregnancy?”. Field 4041 was collected from women who indicated that a doctor had told them they had diabetes during pregnancy (1061 cases). We additionally used data from self-reported illnesses category on gestational diabetes (data-field 20002, code 1221) (249 cases), and hospital in-patient episode data with diagnosis code O24.4 “Diabetes mellitus arising in pregnancy” (213 cases) (“Diagnoses—main ICD10”) (data-field 41270). Altogether, we have 1270 cases of gestational diabetes.

The control group contains women who were pregnant and gave live births but did not report gestational diabetes, gestational hypertension/preeclampsia, or recurrent pregnancy losses. Furthermore, women with preexisting conditions were excluded from the control group. Specifically, for the control pool, we used data for women who had at least one live birth without complications for gestational diabetes, gestational hypertension/preeclampsia, eclampsia, or pre-existing diabetes. We included women with the UKBB diagnosis codes related to live birth and pregnancy O2–O9 or Z34.8, Z37.0, Z37.2, Z37.3, Z37.5, Z37.6, Z38.1, Z38.3, Z38.6, Z39 (data-field 41270); but excluding those related to codes relevant for gestational diabetes, gestational hypertension, eclampsia (codes O10–O16), and preexisting diabetes (O24.0, O24.1, O24.2, O24.3, O24.9). Overall, the procedure resulted in the control set comprising 13,400 women.

### 2.2. Genotype and Phenotype Data

To identify variants for building the PGS we utilized the results from Neale lab GWAS of UKBB phenotypes www.nealelab.is/uk-biobank/ (accessed on 9 June 2022). We combined the results from traits related to GDM self-report diagnoses (data-field 4041 and data-field 20002, code 1221) and selected SNPs below the significance cutoff $1\times 10^{5}$. Overall, this analysis yielded 120 distinct SNPs. The list of relevant SNPs was further extended based on published GDM studies [15,16,17] resulting in a final set of 174 SNPs considered in the analysis.

In addition to the genotype data, we utilized the data on participants’ body mass index (BMI) to investigate the relationship between the genetic risk of GDM and BMI. In cases where participants’ BMI (data-field 21001) was repeatedly assessed over the years, the most recently reported BMI was taken as a BMI estimate. Individuals whose BMI was not reported or was very low (below 18.5) were excluded from the BMI analysis.

### 2.3. Procedure for Learning the Polygenic Risk Scores

A PGS is derived from a list of relevant SNPs. PGS is a risk-weighted sum of the genetic variants, where the number of effect alleles is represented by either 0, 1, or 2, and the weights are identified by a machine-learning model. The SNPs were first clumped using PLINK’s LD-based clumping procedure with the physical distance threshold for clumping set to 10,000, r2 threshold set to 0.02, and the EUR population from the “1000 genomes” project used as a reference population. The SNPs absent from the reference dataset were manually checked for LD. The described clumping procedure resulted in 94 unique SNPs used in further modeling. To further account for potential collinearity among the predictor variables, the variance inflation factor (VIF) score was calculated for each SNP retained after clumping. SNPs whose VIF was higher than 10 [18] were iteratively removed from the set until all VIF values were below the said threshold. To balance the number of cases and controls in our machine learning, controls were randomly sampled (10 times) so that the number of controls is 4-times bigger than the number of cases. Thus, this procedure yielded ten different datasets for learning the models.

Next, two modeling methods were utilized to determine the weights for each variant. The first procedure relies on the generalized linear model in R statistical language that fits a logistic regression model to cases and controls. More specifically, the trainControl and train functions from R’s caret package were used to fit the models to the data. The models’ performance was estimated by repeating the 10-fold cross-validation process ten times. Finally, once the ten models were trained (i.e., each of the ten datasets was used to train a model), the best model was selected based on the area under the receiver operating characteristic curve (AUC). The second procedure also aimed at fitting a logistic regression model to the data but using a forward-selection method that minimizes the amount of information loss due to the model’s simplification, i.e., the Akaike Information Criterion. For this, we used the stepAIC function in MASS and car R packages. When learning the models on each of the ten datasets, the data was separated into training and test sets to enable performance estimation. Again, the best-performing model was selected based on the estimated AUC.

95% confidence intervals (CI)s for odds ratios (OR) were calculated as Wald intervals (or Normal approximation intervals) using the oddsratio function from the epitools package in R.

### 2.4. Mendelian Randomization

To run Mendelian Randomization analyses we used the TwoSampleMR package in R and utilized summary-level data for the genetic associations with exposure and outcomes provided as part of the package. For the outcome, gestational diabetes from Finnish Gestational Diabetes [19] study was used. For exposure, BMI, waist circumference, hip circumference, glycaemic traits (glucose, glycated hemoglobin) were obtained from the MR-Base GWAS catalog [20]. Females-specific waist-to-hip ratio (WHR) and four top body principal components (anthropometric measures) are downloaded from Zenodo [21]. Genetic instruments associated with exposures were obtained with the significance threshold $1\times 10^{-8}$. Pleiotropy was evaluated based on the intercept calculated by MR-Egger regression using mr_pleiotropy_test with *p*-value threshold p=0.05. We report exposure-outcome relationships that change by at least 10% in the odds ratio (OR>=1.1 or OR<=0.9).

### 2.5. SNP Annotation

SNPs are annotated with genes and genome-wide association studies (GWAS) using SNPnexus, a web-based variant annotation tool [22,23]. Functional analysis on gene level is performed using Functional Mapping and Annotation of Genome-Wide Association Studies, FUMA [24].

## 3. Results

### 3.1. Polygenic Risk Score

We here construct a dataset of cases (1270) for GDM and controls (13,400) from the UKBB, and perform a case-control retrospective study using data. As our goal is to develop a screening tool to identify at-risk group for GDM, we combined those women who had only diabetes during pregnancy, and those who have later developed other types of diabetes (see Section 2: "Materials and Methods") for detailed explanation of selection of cases and control groups).

PGS was calculated as a weighted sum of 174 genetic variants selected as described in the Methods. Weights for each variant were learned by utilizing a generalized linear model with added collinearity analysis for the predictor variables. The best-performing model was selected based on the estimated AUC (for details, see Section 2: "Materials and Methods"). Resulting PGS model has 84 SNPs (Table 1 and Supplementary Table S1) with AUC = 0.64. We also used the stepwise (forward-selection) procedure that resulted in 51 SNPs (Supplementary Table S1) and slightly lower AUC = 0.63. We further discuss results from the first model in the paper, and provide results for the step-wise model in the Supplementary Tables.

**Figure 1:**
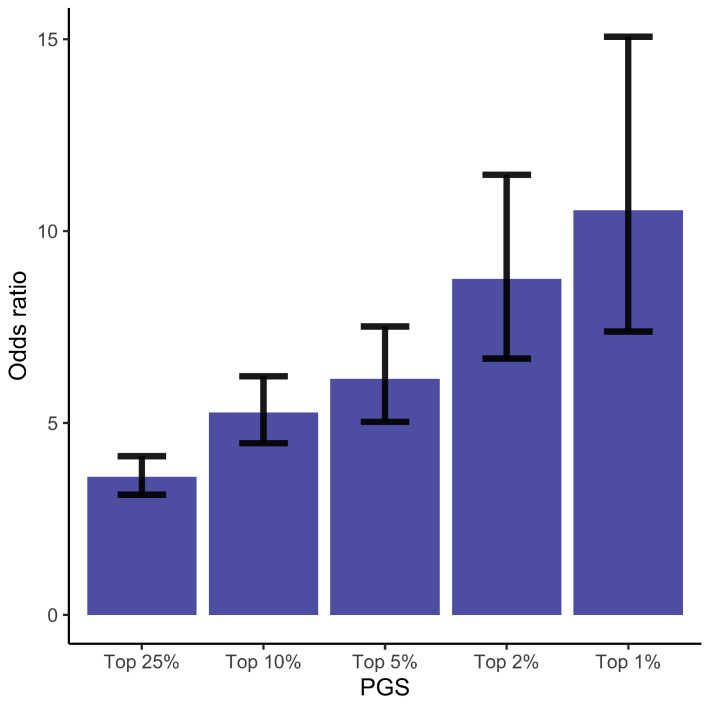
Odds Ratios and the corresponding 95% confidence intervals for the GDM. The odds of being diagnosed with GDM for individuals ranked in the top 1%, 2%, 5%, 10%, and 25% of the PGS compared to the odds of developing GDM in the lower 50% of the PGS. See also Supplementary Table S2.

To identify women at high risk of GDM, we computed odds ratios (ORs) for GDM by contrasting the individuals ranked in the top 1%, 2%, 5%, 10%, and 25% PGS values to the individuals whose PGS values are in the lower 50%. Compared to women in the lowest half of the PGS, women in the top quantile have OR = 3.60 (CI = [3.13–4.14]), for the top 10% OR = 5.27 (CI = [4.47–6.22]), for the top 5% OR = 6.15 (CI = [5.03–7.52]), for the top 2% OR = 8.75 (CI = [6.68–11.47]), and for the top 1% OR = 10.55 (CI = [7.38–15.06]) (Figure 1 and Supplementary Table S2). Here CI stands for 95% confidence interval. Similar results are obtained for a step-wise model (Supplementary Table S2).

Thus, the developed PGS can be utilized as an early screening tool as it can predict women at high risk of GDM before they become pregnant, and hence allows for early lifestyle changes and close monitoring.

### 3.2. GDM Risk and BMI

Many observational studies have already reported that being overweight is the strongest predictor of GDM [10], while obesity has been concretely established as a mediator of chronic, low-grade, systemic inflammation [25,26]. Genes implicated in BMI in earlier GWAS are significantly over-represented (p= $1.37\times 10^{-7}$ in genes that annotate SNPs from our PGS model) (Supplementary Table S3).

**Figure 2:**
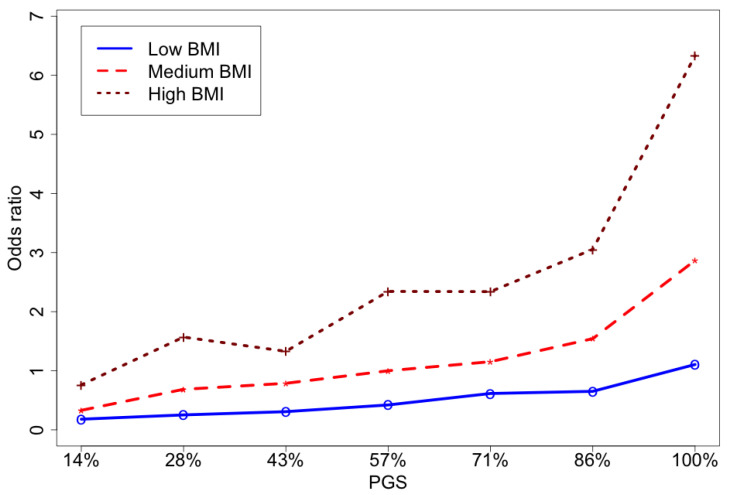
The odds ratios of the groups defined based on BMI and PGS levels. The group of participants with medium-level PGS (43–57%) and medium level BMI (25–30) are taken as a reference group (Supplementary Table S2).

To further investigate the association of BMI within genetic risk groups with GDM, we divided samples into three groups according to BMI: low (18.5–25), medium (25–30), and high >=30 [27]. Furthermore, the PGS was divided into seven levels (i.e., septiles). In this manner, the participants were separated into 21 groups based on their similar BMI and PGS. Computed ORs for each group were then compared to those with the medium BMI group and median PGS (Figure 2 and Supplementary Table S2). Similar results are obtained for a step-wise model (Supplementary Table S2).

Across all three BMI groups, higher PGSs were associated with higher incidences of GDM. The effect of genetics in the low BMI group was very modest while in medium and high BMI groups the risk of GDM was increasing at least linearly with percentile of PGS. High BMI was associated with much higher risks even compared to high PGS with medium and low BMI. Thus, our studies confirm that the contribution of BMI to the risk of GDM is substantial, and it outweighs the contribution of genetics for low, and even medium BMIs.

It is worth noting that for most of the cases and controls in our dataset, reported BMI is measured years after pregnancy and the occurrence of GDM. The age of UKBB participants is 37–73 with the mean age 56.53. Hence, it is not possible to dissect the cause and effect here. This data does not explain whether GDM may have triggered diabetes that resulted in higher BMI later in life, or pre-pregnancy high BMI is a risk factor for GDM.

To resolve this, we turn to Mendelian Randomization (MR), an increasingly popular computational technique often referred to as “nature’s randomized trial”. MR uses genetic instrumental variables to make causal inferences between exposures and outcomes [28]. Earlier MR analyses investigated causal effects on GDM of 282 metabolic measures and risk factors available in the MR-Base GWAS catalog [20], including metabolites, anthropometric measures, hormones, immune system phenotypes, kidney traits and metals [12]. They reported that only BMI demonstrated significant evidence for a causal effect on GDM risk.

### 3.3. GDM Risk and Female-Specific Anthropometric Measures

We performed two-sample MR analyzes to investigate causal effects of BMI, waist circumference, other anthropometric measures, and glycemic traits on GDM from Finnish Gestational Diabetes (FinnGeDi) [19]. Our MR analyses confirm that genetically proxied BMI (ukb-b-19953) significantly and causatively increases the risk of GDM (OR = 1.73; CI = [1.51–1.98]; p= $3.63\times 10^{-15}$) (Supplementary Table S4). Similarly, genetically proxied waist circumference (ieu-a-62) increases the odds of GDM by more than 2-fold (OR = 2.38, CI = [1.57–3.61]; p= $4.31\times 10^{-5}$). Similar increase in the risk of GDM is caused by hip circumference (ieu-a-51). The estimated causal effect of BMI, waist circumference, hip circumference on GDM risk was consistent for different exposure variables, *p*-value cut-offs and across multiple MR models.

We further utilized female specific measures, such as waist-to-hip ratio (WHR) and four specific anthropometric measures (axes) computed from fourteen anthropometric traits from the UK Biobank through principal component analysis [21]. The top four principal components were defined as new anthropometric measures representing body size, adiposity, predisposition to abdominal fat deposition, and lean mass.

Female specific waist-to-hip ratio (WHR) is the top anthropometric risk factor for GDM (OR = 1.76, CI = [1.51–2.06]; p= $2.77\times 10^{-12}$). Further, female specific adiposity (OR = 1.71, CI = [1.46–2.01]; p= $5.44\times 10^{-11}$) and predisposition to abdominal fat deposition (OR = 1.44, CI = [1.28–1.63]; p= $2.43\times 10^{-9}$) are also significantly associated with the odds of GDM. It was reported that adiposity had much stronger effects on many obesity-related diseases, including diabetes, hypertension, hypercholesterolemia and ischemic heart disease [21]. Similarly, predisposition to abdominal fat deposition, despite being weight- and BMI-neutral, was a risk factor for many of the same obesity-related diseases as adiposity (Supplementary Table S4).

MR analyses further confirm that genetically proxied levels of glycemic traits such as glucose (ieu-b-114; OR = 5.98, CI = [2.80–12.73]; p= $3.61\times 10^{-6}$), and glycated hemoglobin levels (ebi-a-GCST90002244; OR = 4.74, CI = [1.82–12.32]; p=0.0014) causatively and substantially increase the odds of GDM (Supplementary Table S4). This is expected as glycemic traits are used to define GDM, and earlier studies reported that genetic risk scores for elevated fasting glucose and insulin, reduced insulin secretion and sensitivity have been used to predict GDM risk, with and without adjustment for body mass index (BMI) and maternal age [14]. We further identify genetically proxied insulin-like growth factor 1 (IGF1), implicated in glucose homeostasis, as a causative factor for GDM (OR = 1.15; CI = [1.04–1.29]; p=0.009). A longitudinal study [29] observed a significantly increased risk of GDM associated with higher concentrations of IGF-I (as well as molar ratio of IGF-I to IGFBP-3, and lower concentrations of IGFBP-2), weeks earlier before GDM is typically screened for.

## 4. Discussion

Women who are at average risk of GDM are currently recommended an oral glucose tolerance test between 24 and 28 gestational weeks as the method of GDM diagnosis. According to the Mayo clinic, women at high risk of GDM are generally determined by being overweight before pregnancy, and having diabetes in the family. Women at high risk may be offered a test for GDM early in pregnancy, likely at the first prenatal visit.

There is an obvious problem with this approach. GDM carries significant short-term and long-term adverse health outcomes for both mother and offspring, which reinforces the significance of understanding risk factors, in particular modifiable factors, for GDM and of preventing the condition. Treating the short- and long-term complications of GDM are costly, amounting to tens of thousands of USD per person. Therapeutic options for women with GDM are limited to insulin injections or a small selection of second-line oral antihyperglycemic agents. Clearly, current approaches do not address preconception care and lifestyle interventions that might prevent, control or mitigate risks associated with GDM.

In this study we develop and cross-validate a genetics-based screening tool for identifying women at risk for GDM even before they become pregnant. From a saliva or a cheek swab test, a PGS, based on 84 genetic variants, predicts that women in the top 5% of PGS have a more than 6-fold (OR = 6.15; CI = [5.03–7.51]) increased risk of gestational diabetes compared to lower 50% of the PGS.

### 4.1. Functional Analysis

Out of 84 SNPs utilized in the PGS, 37 have been implicated in various GWAS with the most prevalent traits being associated with diabetes, glucose, and glycemic traits (Supplementary Table S3). Most of the annotated SNPs have been implicated in multiple phenotypic traits, with the top pleiotropic SNP, rs1260326 in the GCKR gene being associated with 113 traits, from diabetes, glucose and glycemic pregnancy traits to anthropometric traits and various biomarkers.

Just over a third of SNPs (31) from the PGS are annotated to genes (Supplementary Table S3), most of which are highly pleiotropic. Functional enrichment analysis of these genes results in numerous phenotypic traits and biological processes that are significantly over-represented (Supplementary Table S3). Top highly enriched categories include fasting blood glucose (p= $2.46\times 10^{-33}$), type 2 diabetes (p= $2.04\times 10^{-27}$), glycated hemoglobin levels (p= $2.88\times 10^{-19}$), glycemic traits in pregnancy (p= $2.83\times 10^{-11}$), and other glucose and insulin related traits and biological processes such as cell signaling, hormone secretion and transport. Other over-represented traits include biomarkers, such as triglycerides (p= $1.07\times 10^{-12}$), HDL cholesterol (p= $1.45\times 10^{-10}$), C-reactive protein levels (p= $4.28\times 10^{-7}$).

Over half of SNPs (47) from the PGS do not have GWAS annotations, and 24 of these SNPs are located in the intergenic regions, not mapped to any coding genes or non-coding RNAs. Four SNPs that contribute to the PGS are mapped to the X chromosome, and only one of these SNPs (rs5945326) has been implicated in type 2 diabetes in Europeans and East Asians. The molecular events underlying the effect of 3 other SNPs on the development of GDM are not known.

### 4.2. Risk Factors for GDM

We further identified anthropometric measures that causally increase the risk of GDM which is in line with earlier observations from observational studies. Specifically, BMI, WHR, adiposity, and abdominal fat deposition are significantly associated with an increased risk of GDM. Interestingly, the abdominal fat deposition, despite being weight and body-mass neutral, is a significant risk factor for GDM with a slightly weaker effect (OR = 1.4) compared to the contribution of WHR (OR = 1.75) or adiposity (OR = 1.7). Predisposition to abdominal fat deposition, likely reflecting a shift from subcutaneous to visceral fat, has already been identified as a risk factor for ischemic heart disease, hypercholesterolemia, and diabetes. We here confirm that predisposition to abdominal fat deposition is a risk factor for GDM that needs to be taken into consideration while assessing women’s risk.

### 4.3. Limitations

Our model has several limitations. Firstly, the PGS screening tool developed in this study has a moderate AUC of 0.64. However, we demonstrate that the PGS potentially captures sufficient information to identify a high-risk subgroup of women who could be offered lifestyle modifications and closer monitoring during or even before their pregnancy. In fact, there have been lots of discussions on utilizing PGSs as predictive biomarkers for high-risk subgroups for a wide range of diseases, including cancers [30]. Hence, PGS developed in this paper can be used as an early predictive compound biomarker for GDM.

Another limitation of this study is the fact that it was built on data from the UKBB which largely contains a white European population. Hence, its applicability to other ethnic groups may be compromised. Future studies should include women from other ethnic groups, and in particular Black and Hispanic women who are disproportionally affected by GDM.

Further, while our study quantifies the odds ratios for several risk factors, our screening tool does not combine them into one model to provide step-by-step guidance for clinicians. The model should also include the mother’s age as several observations report advancing pregnancy age as a risk factor for GDM. This is a subject of further studies that require large cohorts from different ethnicities.

## 5. Conclusions

In this study, we develop a genetics-based predictive screening tool for GDM. This inexpensive test can be seamlessly utilized at home or in clinical practice to identify high-risk women even before they become pregnant providing an opportunity to offer them GDM preventative preconception lifestyle strategies, and close monitoring by healthcare providers during the early stages of pregnancy. This early screening tool for GDM can potentially be integrated with other risk factors, including anthropometric measurements, and biomarkers.

## Figures and Tables

**Table 1:** SNPs and Weights in the Polygenic Risk Score Model Table ^1^.

Term	Estimate	Std. Error	Statistic	*p* Value	Overlapped Gene	Nearest Upstream Gene	Nearest Downstream Gene
rs10830963	0.267	0.046	5.834	$5.40\times 10^{-9}$	MTNR1B		
rs6959526	0.378	0.081	4.671	$2.99\times 10^{-6}$	MGAM		
rs34075917	0.205	0.045	4.54	$5.63\times 10^{-6}$		CTC-419K13.1	ENC1
rs7903146	0.204	0.046	4.45	$8.60\times 10^{-6}$	TCF7L2		
rs11257655	0.209	0.050	4.186	$2.84\times 10^{-5}$		RN7SL232P	RN7SL198P
rs4746822	0.190	0.046	4.123	$3.74\times 10^{-5}$	RP11-227H15.4; HKDC1		
rs79953201	0.583	0.144	4.037	$5.42\times 10^{-5}$	HAPLN1		
rs34882181	−0.149	0.042	−3.512	$4.00\times 10^{-4}$	PTPRD		
rs535447438	−0.155	0.046	−3.355	$8.00\times 10^{-4}$	LPHN2		
rs141240229	0.318	0.096	3.296	0.001		EEF1A1P9	AC004066.2
rs116847631	0.202	0.062	3.279	0.001	PGR		
rs7957197	−0.180	0.058	−3.127	0.0018	OASL		
rs116966095	0.258	0.085	3.04	0.0024	CFDP1		
rs62603092	−0.248	0.082	−3.014	0.0026		RP11-274K13.5	snoU13
rs2866307	−0.145	0.049	−2.939	0.0033		RP11-168E17.1	RNU6-578P
rs7743373	−0.152	0.052	−2.902	0.0037		RP3-435K13.1	RP3-455E7.1
rs340874	0.123	0.043	2.846	0.0044	PROX1-AS1; PROX1		
rs62052363	0.251	0.092	2.735	0.0062	PKD1L2		
rs568927434	0.134	0.049	2.734	0.0063	SPP1		
rs4376068	0.121	0.045	2.712	0.0067	IGF2BP2		
rs62170385	0.270	0.102	2.64	0.0083	ARHGAP15		
rs174550	−0.120	0.046	−2.588	0.0096	FADS2; FADS1		

^1^ Table with top most significant SNPs from the polygenic risk score as the result of machinelearning procedure. SNPs are annotated using SNPnexus. Full Table with all 84 SNPs and their weights is available in Supplementary Tables S1 and S3.

## Data Availability

The results presented in this study are available in Supplementary Material. Restrictions apply to the availability of analyzed data sets; UK Biobank data is available after completing an application procedure.

## Supplementary Materials

The following supporting information can be downloaded at: https://www.mdpi.com/article/10.3390/jpm12091381/s1, Table S1: Linear PGS models for GDM; Table S2: Odds ratios for the GDM; Table S3: Annotations of SNPs; Table S4: Two Sample MR.
